# Pregnancy outcomes in women with infertility and coexisting endometriosis and adenomyosis after laparoscopic surgery: a long-term retrospective follow-up study

**DOI:** 10.1186/s12884-021-03851-0

**Published:** 2021-05-18

**Authors:** Jinghua Shi, Yi Dai, Junji Zhang, Xiaoyan Li, Shuangzheng Jia, Jinhua Leng

**Affiliations:** 1 Department of Obstetrics and Gynecology, Peking Union Medical College Hospital, Chinese Academy of Medical Science & Peking Union Medical College, Beijing, P. R. China; 2 National Clinical Research Center for Obstetric & Gynecologic Diseases, Beijing, P. R. China; 3 Permanent address: Department of Obstetrics and Gynecology, Peking Union Medical College Hospital, Beijing, 100730 P. R. China

**Keywords:** Adenomyosis, Endometriosis, Infertility, Laparoscopic surgery, Pregnancy outcome

## Abstract

**Background:**

Adenomyosis (AM) and endometriosis (EM) often coexist. Laparoscopic surgery is one of the main methods for diagnosing and treating these conditions. This study aims to investigate the pregnancy outcomes of women with infertility with both AM and EM after laparoscopic surgery and to identify the relevant associated factors.

**Methods:**

This is a retrospective study involving women with infertility diagnosed with EM and AM. All patients had undergone laparoscopic surgery and were divided into two groups according to their pregnancy outcomes. Demographic data, operation records, and pregnancy outcomes were collected.

**Results:**

A total of 226 female patients with infertility diagnosed with both AM and EM underwent laparoscopic surgery. Of these, a total of 176 patients completed follow-up. Ninety-seven patients had live births, including 81 full-term and 16 preterm deliveries. The clinical pregnancy and live birth rates were 67.4 and 55.11%, respectively. One hundred thirty-five patients received in vitro fertilization (IVF), with 70 (51.85%) of these patients having live births. Age, endometrioma size, and uterus size were significantly lower in those who had a successful delivery. There was no statistically significant difference in symptoms, except that those who achieved live birth had a lower rate of anaemia (13.40% vs. 25.32%, *p* = 0.044). The group that did not proceed to have a live birth had a higher percentage of ovarian and peritoneal endometriosis (*p* < 0.05), while the distribution of deep infiltrating endometriosis and adenomyosis types were similar. Mean uterus diameter (OR: 0.636, 95% CI: 0.434–0.932, *p* = 0.020) and endometriosis fertility index (EFI) (OR: 1.299, 95% CI: 1.101–1.531, *p* = 0.002) were significantly correlated with live birth in the multivariable analysis.

**Conclusions:**

Endometriosis and adenomyosis appear to have an adverse effect on pregnancy outcome. These might be related to the size of the uterus and EFI. Obstetricians and gynaecologists should be alert to this potential adverse effect and manage these patients accordingly.

## Background

Adenomyosis (AM) and endometriosis (EM) [1] are benign conditions of the uterus, defined by the presence of endometrial glands and stroma within the myometrium and outside of the uterus, respectively. Histological diagnosis is the most accurate method for identifying these conditions, but they can also be diagnosed clinically [2, 3] with imaging modalities when patients present with symptoms. These symptoms include dysmenorrhea, dyspareunia, abnormal uterine bleeding, and infertility. AM shares some pathogenic mechanisms with EM [4]. Our previous study reported that 39.9% of women with endometrioma also had AM [5], while 33.3% [6] of 72 patients histologically diagnosed with AM had concomitant EM. Chapron et al. [7] reported that the coexistence percentage could be as high as 87.4%.

AM and EM both have adverse effects on fertility, complicating treatment [8, 9]. In addition to impacts on reproductive performance (infertility and spontaneous pegnancy loss), pregnancy outcomes (preterm labour, foetal growth restriction, placenta previa and even uterine rupture) are also affected by AM [10, 11] and EM [12].

Laparoscopic surgery is one of the primary ways [13] to diagnose and treat these conditions, but difficulty remains in predicting pregnancy outcomes in women with infertility diagnosed with both AM and EM. The endometriosis fertility index (EFI) has been proven to be a useful model to predict pregnancy outcomes [14]; however, it does not include adenomyosis. Additionally, the effect of the type of coexisting adenomyosis on fertility outcomes has not been fully evaluated, with few studies specifically concentrating on this topic. In this study, we aimed to explore pregnancy outcomes through long-term follow-up and analyse the factors related to fertility outcomes of women with infertility with both AM and EM after laparoscopic surgery.

## Methods

### Ethical approval and informed consent for study

This study was approved by the Ethics Committee (institutional review board of Peking Union Medical College Hospital, No. S-K1055), and all procedures involving human participants followed the ethical standards of the institutional review board (IRB) from the study centre. Informed verbal consent was obtained from all patients at their follow-up interviews.

### Subject selection

We identified 257 patients who underwent surgery for the first time in our hospital with a diagnosis of both EM and AM between January 2013 and December 2017. Among them, 226 (87.94%) patients with infertility underwent laparoscopy and who wished to conceive after surgery were followed-up for a minimum of 2 years after the operation. The exclusion criteria were as follows: (1) age < 20 or > 40 years; (2) bilateral oophorectomy or hysterectomy; (3) intraoperative conversion to laparotomy; and (4) concomitant diseases that clearly affect fertility, such as submucosal fibroids, premature ovarian failure, systemic lupus erythematosus, malignant tumours, polycystic ovary syndrome, male factor and reproductive malformation.

### Clinical definitions

Infertility [15] was defined as the inability to conceive despite frequent, unprotected sex for at least one year, and a successful delivery was defined as the patient having a live birth. Primary infertility referred to a couple without a pregnancy history, while secondary infertility was used for couples who have had at least one conception.

Ultrasonographic exams were performed after the menstrual cycle immediately prior to surgery. Features on ultrasound that suggest AM were as follows: asymmetrical myometrial thickening, myometrial cysts, linear striations, hyperechoic islands, or irregular and thickened endometrial–myometrial junctional zones [16]. If more than two features were present and the lesions were located in only one part of the uterine wall, a diagnosis of focal AM was made. Diffuse AM was diagnosed for lesions in more than one site within the myometrium, more often being widely spread rather than forming a confined lesion [17]. Endometriosis was visually inspected by laparoscopy and histologically confirmed. Deep infiltrating endometriosis was diagnosed as the presence of one or more endometriotic nodules infiltrating deeper than 5 mm [18].

### Surgical intervention, variables and measurements

During the surgery, endometriomas were removed, and peritoneal endometrial tissue was treated with bipolar electrocoagulation. Deep infiltrating endometriotic nodules and adenomyoma lesions were resected after consultation between doctors and the patient, especially for those who had severe symptoms or repeated in vitro fertilization and embryo transfer (IVF-ET) failure. Pre-surgical symptoms and surgical data, including postoperative complications, were retrieved from patient admission and operative databases. The revised American Fertility Society (rAFS) score, staging from the revised American Society for Reproductive Medicine (rASRM), and EFI [19] were collected according to surgical records and infertility history. Post-surgical symptoms and pregnancy outcomes were collected from follow-up interviews with outpatients.

In this study, independent variables were classified into three groups: demographic factors, including age, body mass index (BMI), history of abortion and assisted reproductive therapies (ART), menstrual cycle length, dysmenorrhea, anaemia, CA125 and follicle stimulating hormone (FSH). Surgical factors included mean uterus diameter, mean cyst diameter, type of EM and AM, blood loss, surgical time, resection of AM and deep infiltrating endometriotic (DIE) nodules, rAFS score and EFI. Medical treatment and IVF after surgery were also examined as potential variables related to pregnancy outcome. The dependent variable for all these was live birth.

### Statistical analysis

Continuous data are presented as the median (interquartile range) or mean ± SD and were compared using the t-test or ANOVA. Categorical data are described by the number of patients (including percentages) and were compared with Fisher’s exact test or the chi-square test. Variables identified with univariable analysis with a *P* value less than 0.2 were subjected to multiple logistic regression (stepwise). Odds ratios (ORs) and 95% confidence intervals (CIs) were calculated as measures of the impact of the variables on live birth. All analyses used a two-tailed α of 0.05 and were performed using SPSS software (Version 20.0, IBM Corp., Armonk, NY, USA), and *p* < 0.05 was considered significant.

## Results

### Patient characteristics

**Fig. 1:**
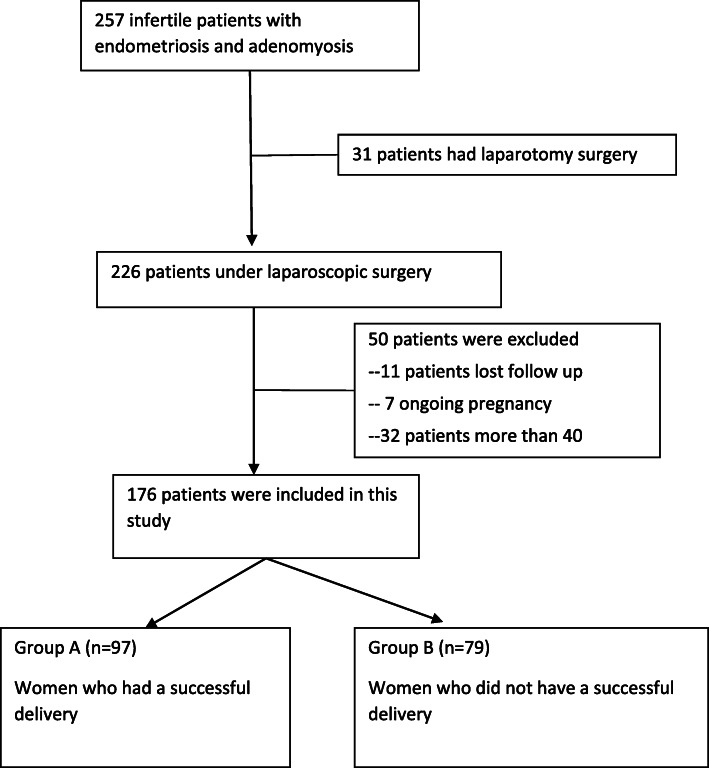
Flowchart of patient selection

During the study period, 226 patients with infertility with both AM and EM were identified and underwent laparoscopic surgery. Forty-three patients were excluded due to either age criteria (*n* = 32) or insufficient follow-up data (*n* = 11). Seven patients who became pregnant did not deliver and were thus also excluded. To report the factors associated with successful live births, we divided the 176 remaining women into two groups: Group A (*n* = 97) contained 81 women who give birth to full-term infants and 16 who birthed preterm infants, and Group B (*n* = 79) consisted of those patients who did not achieve a successful delivery. The average BMI (kg/m^2^) was 21.17 ± 3.86. A flowchart describing the group selection process for the patients is provided in Fig. 1. The basic characteristics of both groups are presented in Table 1.

**Table 1:** Population characteristics of the live birth and non-live birth groups

	Group A	Group B	*p* value
	Women who had a successful delivery(*n* = 97)	Women who did not have a successful delivery(*n* = 79)	
Age (years)	32.52 ± 3.20	33.53 ± 3.95	0.046*
BMI (kg/m2)	21.74 ± 2.78	20.51 ± 4.72	0.033*
Type of Infertility n (%)			NS
Primary infertility	60 (61.86%)	52 (65.82%)	
Secondary infertility	37 (38.14%)	27 (34.18%)	
Gravida	0 (0–5)	0 (0–5)	NS
Parity	0 (0–1)	0 (0–2)	NS
Abortion history	19 (19.59%)	23 (29.11%)	NS
Previous ART	21 (21.65%)	14 (17.72%)	NS
Duration of infertility (years)	3 (1–8)	2 (1–11)	NS
Length of period (day)	5.88 ± 1.86	6.22 ± 2.04	NS
Menstrual cycle (day)	28.94 ± 6.17	28.94 ± 9.35	NS
Duration of dysmenorrhea (years)	5.33 ± 5.77	6.30 ± 6.29	NS
Dysmenorrhea (VAS)	4.22 ± 3.59	4.58 ± 3.49	NS
Anemia	13 (13.40%)	20 (25.32%)	0.044*
CA125(U/ml)	80.67 ± 92.47	109.57 ± 98.48	NS
FSH (IU/l)	7.12 ± 2.21	8.43 ± 2.81	0.005*
E2(pg/ml)	53.69 ± 41.14	48.88 ± 21.85	NS
Mean uterus diameter (cm)	4.91 ± 0.79	5.42 ± 1.20	0.001*
Mean cyst diameter (cm)	2.63 ± 3.43	4.38 ± 4.00	0.002*

Note: Data are presented as mean ± SD, median (interquartile range) or *n* (%); NS, not significant; BMI, body mass index; ART assisted reproductive technology; VAS, visual analog scale; CA125, cancer antigen 125; FSH, follicle stimulating hormone; E2, estrogen

*p < 0.05

### Surgical findings

Compared with the women who did not have a successful delivery, the women who had a successful delivery had significantly lower rAFS scores (33.62 ± 33.53 vs. 47.56 ± 38.22, *p* = 0.011) and higher EFIs (6.87 ± 2.04 vs. 5.67 ± 2.09, *p* = 0.001). Blood loss during laparoscopy in group A was also less than that in group B (46.03 ± 51.80 ml vs. 84.08 ± 114.39 ml, *p* = 0.009), as was surgical time (56.60 ± 21.42 min vs. 65.76 ± 27.34 min, *p* = 0.022). Seventy-six patients underwent adenomyosis/adenomyoma resection. We also found that the non-live birth group had a significantly higher proportion of ovarian endometriosis (OEM) and peritoneal endometriosis (PEM) than the live birth group. However, the differences in DIE, AM types, leiomyoma, endometrial polyps, or obstruction of the oviduct between the two groups were not significant (Table 2).

**Table 2:** Surgical findings between the live birth and non-live birth groups

	Group A(*n* = 97)	Group B(*n* = 79)	*p* value
Ovarian endometriosis	49 (50.52%)	57 (72.15%)	0.004*
Peritoneal endometriosis	65 (67.01%)	40 (50.63%)	0.028*
Deep infiltrating endometriosis	35 (36.08%)	22 (27.85%)	NS
AM type			NS
Diffuse	52 (53.61%)	43 (54.43%)	
Local	45 (46.39%)	36 (45.57%)	
Leiomyoma	29 (29.90%)	18 (22.78%)	NS
Obstruction of oviduct	22 (22.68%)	23 (29.11%)	NS
Endometrial polyps	31 (31.96%)	25 (31.64%)	NS

Note: Data are presented as n (%); NS, not significant

*p < 0.05

### Fertility results and pregnancy outcomes

**Fig. 2:**
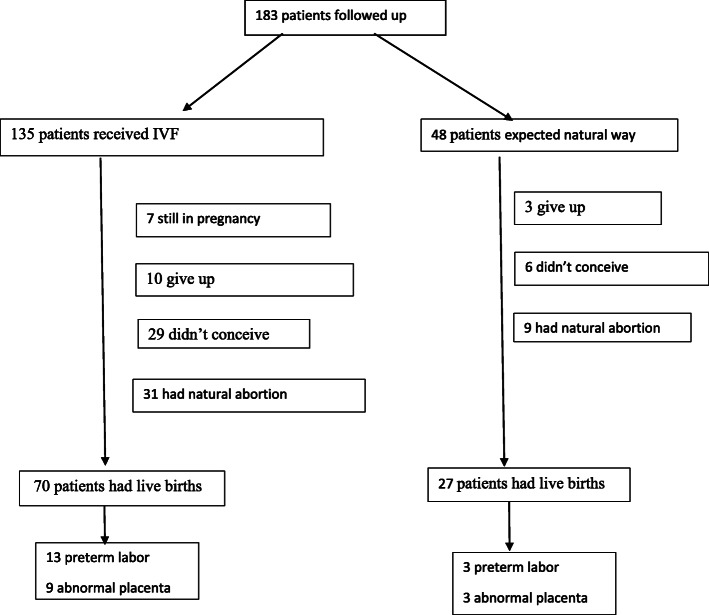
Flowchart of reproductive outcomes in AM and EM patients who underwent laparoscopic surgery

After long-term follow-up (median 47 months, range 24–80 months), 35 (19.13%) patients had failed to conceive, and 13 (7.10%) patients stopped trying to conceive: four due to disease relapses, two due to depression, four due to premature ovarian failures (POFs), one due to endometrial intraepithelial neoplasia (EIN), and two due to repeated IVF failures. One hundred thirty-five (73.77%) patients underwent IVF, and 70 patients had live births (51.85%). The mean time from surgery to pregnancy was 12.89 ± 8.66 months, and the mean time from the first ovulation after surgery to pregnancy was 8.56 ± 8.48 months. The clinical pregnancy rate, or the presence of a foetal heartbeat at 12 weeks of gestation, was 67.4, and 55.11% ultimately achieved a successful delivery. One hundred forty-eight (80.87%) patients had a positive pregnancy test. Forty (27.03%) patients experienced spontaneous pregnancy losses, two (1.35%) had ectopic pregnancies, and five (3.38%) experienced intrauterine deaths (foetal death at or after the 20th week of gestation). Among the women who gave live birth, 16 (16.50%) had preterm labour, and 12 (12.37%) had abnormal placental conditions (seven placenta previa, four placenta acrreta, and one placenta increta). Other detailed information regarding natural/IVF pregnancy outcomes is provided in Fig. 2.

To determine the factors associated with a successful delivery, univariable analysis and multivariable logistic regression analysis were performed. Variables showing a tendency of association with live birth rate (*p* values < 0.20) in the univariable analysis were included in the multivariable model. When two variables were highly correlated, we introduced only one of them into the model and suppressed the other; for example, when examining age and FSH level, the latter was suppressed. Surgical time (*p* = 0.016) and blood loss (*p* = 0.011) showed a tendency of association, but they were excluded because they were highly correlated with the AFS score and type of AM. EFI includes age, infertility type, years of infertility, function of the fallopian tube/fimbria/ovaries, and AFS; therefore, they were not included as candidate factors. EFI (*p* < .001), anaemia (defined as haemoglobin < 110 g/L) (*p* = 0.047), VAS (*p* = 0.112), CA125 (*p* = 0.093), mean uterine diameter (*p* = 0.002), ART (*p* = 0.122), and medical history (*p* = 0.017) were included as covariates in the final model. AM type (*p* = 0.159) and AM surgery (*p* = 0.110) were also included, as they were reported to be related to the pregnancy rate [20]. Table 3 presents the estimated ORs with the standard errors (SE) and 95% CIs between live birth rates and significantly relevant confounders. Other variables demonstrated no significant relationship with live births.

**Table 3:** Significant prognostic factors of live birth rate after multivariable analysis

Characteristics	OR	SE	95% CI	*P* value
Mean uterus diameter	0.636	0.195	0.434–0.932	0.020*
EFI	1.299	0.084	1.101–1.531	0.002*

Note: *OR* odds ratio, *SE* standard error, *CI* confidential interval

*p < 0.05

## Discussion

This study included 176 infertile women with both EM and AM who underwent laparoscopic surgery and subsequently completed long-term outpatient follow-up. To date, only a handful of studies have specially focused on pregnancy outcomes in women with both AM and EM [21–23], while most of the reported studies involved either exclusively EM or AM patients or grouped the two diseases together. Notably, Maignien et al. [24] studied 359 EM patients with infertility and found pregnancy and live birth rates of 44 and 32%, respectively. Our results showed higher rates for both pregnancy and live birth, likely because we recruited only women between the ages of 20 and 40 years, given that age is a potential risk factor for infertility [25]. Another potential reason for the higher pregnancy outcomes in our study than in previous reports might be the low average BMI (21) in our patients. Interestingly, the occurrence of EM pathology has been shown to be inversely correlated with BMI, as obese women with infertility are at lower risk for EM [26]. Here, we documented that the live birth group patients had a slightly but significantly higher BMI and lower rAFS scores than the patients in the non-live birth group, which is in agreement with the finding of Ashraf Moini [27], who showed that BMI was an independent predictive factor of severe endometriosis. However, the exact impact of BMI on pregnancy outcomes in non-obese women with EM has not been fully evaluated to date. Furthermore, the better pregnancy outcomes in our study could be attributed to the frequent and multiple rounds of IVF received by the participants. Among these patients, some underwent IVF immediately after surgery, while some opted for delayed IVF after failing to conceive naturally. Notably, some of our patients were able to have natural live births even after repeated rounds of IVF-ET failure. Considering these potential variabilities, we could not compare the pregnancy outcomes from IVF therapy with those of natural conception.

We observed that EM phenotypes might directly impact pregnancy outcomes, as there were significant differences between the live birth group and the non-live birth group. Our previous results demonstrated the presence of follicles in the cyst walls of endometriomas on histopathological examinations [28], and the FSH level significantly decreases after laparoscopic bilateral ovary endometrioma resection [29]. Vercellini et al. [30] retrospectively analysed 419 patients who naturally conceived based on their EM phenotypes. They reported a total spontaneous pegnancy loss rate of 20.8%, which was much lower than that observed (27.03%) in our study. Moreover, they also noticed a higher spontaneous pegnancy loss rate in women with ovarian endometriomas (26%) than in those with the peritoneal type (12%). However, these findings are in contrast to those in the report by Maignien et al. [24], where the live birth rates per cycle were reported as 13.7, 16.5 and 16% for peritoneal, ovarian, and deep infiltrating EM (*p* = 0.82), respectively.

In addition to the reduced oocyte yield, reduced fertilization rate (FR), and increased spontaneous pegnancy loss rate [31, 32], AM and EM have also been reported to be associated with several obstetrical and foetal complications [12]. However, the evidence is still lacking and contradictory. A recent meta-analysis including 104 reports found that EM could be associated with preterm delivery (OR 1.38, 95% CI 1.01–1.89), caesarean section delivery (OR 1.98,95% CI 1.64–2.38), and neonatal unit admission following delivery (OR 1.29, 95% CI 1.07–1.55) [33]. Two other systematic reviews failed to draw conclusions on obstetrical complications related to EM except for preterm delivery [34] and placenta previa [35]. This study found a preterm birth rate of 16.50% and a placenta previa incidence of 7.22%, which are slightly higher than those in Benaglia L’s report [36]. In this retrospective, matched, case-control study, there was no significant difference in the preterm birth rate between the EM and non-EM groups (14 and 14%, respectively, *p* = 0.89), while placenta previa was found to be more common in women with EM than in the control group (6% versus 1%, respectively; *p* = 0.006, OR 4.8, 95% CI: 1.4–17.2). In our study, the preterm birth rate was 16.50%, and the incidence of abnormal placenta (placenta previa, placenta accreta and placenta increta) was 12.37%. A possible explanation for this might be that our patients had both EM and AM. In a multi-centre retrospective questionnaire survey [37] across 65 facilities including 272 pregnant women with AM, it was found that the preterm delivery rate was 24.4%. Several other studies also further analysed the clinical factors that may affect pregnancy outcomes. A retrospective study of 631 women with EM who became pregnant following ART [38] reported that the incidences of preterm delivery or abnormal placental positioning may not increase in stages I–III but can significantly increase in stage IV. Furthermore, Kim and colleagues [39] reported a preterm labour incidence of 24.56% in a retrospective study and found that uterine wall thickness in the second trimester was related to subsequent preterm delivery. Consistent with these findings, we also observed that the size of the uterus was significantly different between the two groups.

The aetiology of infertility is thought to include disordered inflammatory factors such as irregular prostaglandin (PG, particularly PGE2 and PGF2α) and cyclooxygenase 2 (COX-2) production [40], abnormal ER- and PR-mediated signalling pathways associated with progesterone resistance [41], impaired trophoblast invasiveness, and uterine contractility [42]. These can also be related to a failure of the physiologic transformation of the spiral arteries in the inner myometrial segment or junctional zone (JZ). Alterations in the JZ in women with EM and AM can further influence the vascular resistance of JZ spiral arteries at the onset of decidualization [42] and lead to incomplete spiral artery remodelling and a reduction in placental blood flow [43]. The underlying mechanism is highly complex and currently under debate. The influence of coexisting EM and AM on pregnancy is not necessarily the clinically superimposition of that of the individual conditions; rather, these two diseases might interact with each other.

The major strength of this study is the diagnostic approach used for AM and EM. The gold standard for AM diagnosis is based on histopathological analysis, which is an invasive technique with a potential risk of uterine rupture during pregnancy and delivery [44]. Therefore, non-invasive techniques such as ultrasound and MRI are preferred. MRI, with a sensitivity ranging from 78 to 88% and a specificity ranging from 67 to 93% [45], has been traditionally considered more accurate than ultrasound examination. However, with the development of high-quality transvaginal ultrasound (TVUS), a systematic review including 10 studies (1168 records) indicated that the two techniques are comparable [46]. The accuracy of ultrasound in AM diagnosis is high, with a mean sensitivity of 0.72 (95% CI: 0.65–0.79), specificity of 0.81 (95% CI: 0.77–0.85), and area under the curve (AUC) of 0.85 [47, 48]. To minimize the interrater reliability between TVUS technicians, we submitted all raw figures to two experienced ultrasound physicians for review. Furthermore, ultrasound is much cheaper and more convenient than MRI. The gold standard diagnostic examination for EM and its subtypes is based on laparoscopic surgery [2]. However, women referred to our hospital may have had severe forms of either AM or EM or repeated IVF failure, which might introduce potential selection and comparison biases. In addition, the follow-up did not include an analysis of IVF details. Most patients reportedly went to their local ART centre for controlled ovarian stimulation and IVF-ET and could recall few details except the final results. There are several other limitations of this study. First, our study is retrospective in nature. Second, the number of patients was limited; hence, we could not perform subtype analyses according to obstetric complications. Third, other interfering factors were not included in our analyses, such as the excision of DIEs [21], the use of gonadotropin-releasing hormone agonist (GnRHa) [49], and the complete removal of endometriosis lesions [50]. Fourth, this study only included patients who needed laparoscopic surgery. Patients with severe adhesions and a large uterus might instead require laparotomy. Our study shows that the pregnancy outcome is quite different from that following laparotomy (unpublished data). Although dysmenorrhea, menorrhagia, chronic pelvic pain, dyspareunia, and infertility can often occur from this procedure, one-third of women have no such symptoms [45]. The actual effect of AM and EM on the fertility and sterility rates in women could be more widespread and occult.

## Conclusions

Overall, our study suggests that the coexistence of EM and AM has adverse effects on both reproductive performance and the outcome of pregnancy. Age, BMI, and the sizes of endometrioma and the uterus were related to prognosis. IVF is an important technique for improving the pregnancy rate following surgery. These findings may prove useful in developing treatment plans for infertile patients with AM and EM. Further prospective studies with larger sample sizes are required to draw firmer conclusions.

## Data Availability

The datasets used and/or analyses performed during the study are available from the corresponding author upon reasonable request.

## Abbreviations

AM: Adenomyosis
EM: Endometriosis
rAFS: Revised American Fertility Society
rASRM: Revised American Society for Reproductive Medicine
EFI: Endometriosis fertility index
POF: Premature ovarian failures
EIN: Endometrial intraepithelial neoplasia
IVF: In vitro fertilization
VAS: Visual analogue scale
ART: Assisted reproductive technology
SE: Standard error
PG: Prostaglandin
JZ: Junctional zone
TVUS: Transvaginal ultrasound
FSH: Follicle stimulating hormone
AUC: Area under the curve
OR: Odds ratio
95% CI: 95% confidence interval
NS: Not significant
